# Supplementary material for: Three Phages One Host: Isolation and Characterization of Pantoea agglomerans Phages from a Grasshopper Specimen
Source: Int J Mol Sci. 2023 Jan 17;24(3):1820. doi: 10.3390/ijms24031820 (PMC9915841; doi:10.3390/ijms24031820)
Supplement: Supplementary file 1 [file ijms-24-01820-s001.zip › Supplementary materials captions.pdf]

Table S1: Genome annotation and predicted functions of *Pantoea* phage Nifs112 ORFs;

Table S2: Genome annotation and predicted functions of *Pantoea* phage Nufs112 ORFs;

Table S3: Genome annotation and predicted functions of *Pantoea* phage Nafs113 ORFs.;

Figure S1: The maximum-likelihood tree of terminase/terminase large subunit (TerL) amino acid sequences for verification of the studied phage (Nifs112, Nufs112, Nafs113) packaging strategies. Input alignment had 48 sequences with 1417 columns, 1392 distinct patterns 959 parsimony-informative, 320 singleton sites, and 138 constant sites. The tree was built using VT + F + R4 as the best-fit substitution model. Near zero-length branches were collapsed into polytomies. The tree shown is midpoint rooted. Tip labels are colored based on the distinct packaging strategies the phages seen in the tree employ (experimentally verified for most of the phages represented in the tree). The percentage of replicate trees in which the associated sequences clustered together in the ultrafast bootstrap (UFBoot; 1000 replicates) is shown next to the branches for branches having UFBoot support higher or equal to 95%. The tree is drawn to scale, and branch lengths represent the number of amino acid substitutions per site. Tip labels correspond to the phages from which the respective terminase/TerL amino acid sequences were derived and are in the format of "Protein accession|Phage". Colored bars next to the labels indicate the assumed evolutionary distinct TerL clades, which correspond to different phage genome packaging strategies (LDTR stands for long direct terminal repeats, SDTR—short direct terminal repeats). Tip label colors correspond to the annotated TerL clades, except for studied phage TerL sequence labels, which are in black for easier identification.;

Table S4: Descriptions of the selected phage marker protein amino acid sequence dataset, generated MSAs, and features of the trees built.;

Table S5: Overview of the VconTACT2 identified *Pantoea* phage Nifs112, Nufs112, and Nafs113 first-neighbour publicly available complete genome entries (n=103), taxonomy associated with the respective accessions at the time of writing, and their VconTACT2 and VIRIDIC clustering status;

Figure S2: Viridic generated intergenomic distance matrix of the studied *Pantoea* phage Nifs112, Nufs112, and Nafs113 first neighbors;

Figure S3: Easyfig genome nucleotide sequence comparison of *Pantoea* phage Nifs112 and representatives of the *Eracentumvirus* phage genus (*Erwinia* phage vB_EamP-S2, *Erwinia* phage Era103) using BLASTN (top) and TBLASTX (bottom). Genome representations are drawn to scale with the scale bar indicating 3,000 bp. Arrows represent ORFs and point in the direction of transcription. Arrow color coding is based on the functional groups of the respective ORF putative products according to the legend. Gray boxes represent regions of similarity between the genomes and are colored in gradient, with darker shades of gray representing higher region identity;

Figure S4: Five-category Venn diagram showing the results of Roary analysis of the Nufs112 and closely related phage panproteome at (A) 30% protein identity, (B) 90% protein identity;

Figure S5: Easyfig genome nucleotide sequence comparison of *Pantoea* phage Nufs112 and its most close relatives BLASTN (top) and TBLASTX (bottom). Genome representations are drawn to scale with the scale bar indicating 3,000 bp. Arrows represent ORFs and point in the direction of transcription. Arrow color coding is based on the functional groups of the respective ORF putative products according to the legend. Gray boxes represent regions of similarity between the genomes and are colored in gradient, with darker shades of gray representing higher region identity;

Figure S6: Easyfig genome nucleotide sequence comparison of *Pantoea* phage Nafs113 and *Pantoea* phage vB_PagM_LIET2 using BLASTN (top) and TBLASTX (bottom). Genome representations are drawn to scale with the scale bar indicating 3,000 bp. Arrows represent ORFs and point in the direction of transcription. Arrow color coding is based on the functional groups of the respective ORF putative products according to the legend. Gray boxes represent regions of similarity between the genomes and are colored in gradient, with darker shades of gray representing higher region identity.
