# Supplementary figures and images for: Three Phages One Host: Isolation and Characterization of Pantoea agglomerans Phages from a Grasshopper Specimen
Source: Int J Mol Sci. 2023 Jan 17;24(3):1820. doi: 10.3390/ijms24031820 (PMC9915841; doi:10.3390/ijms24031820)

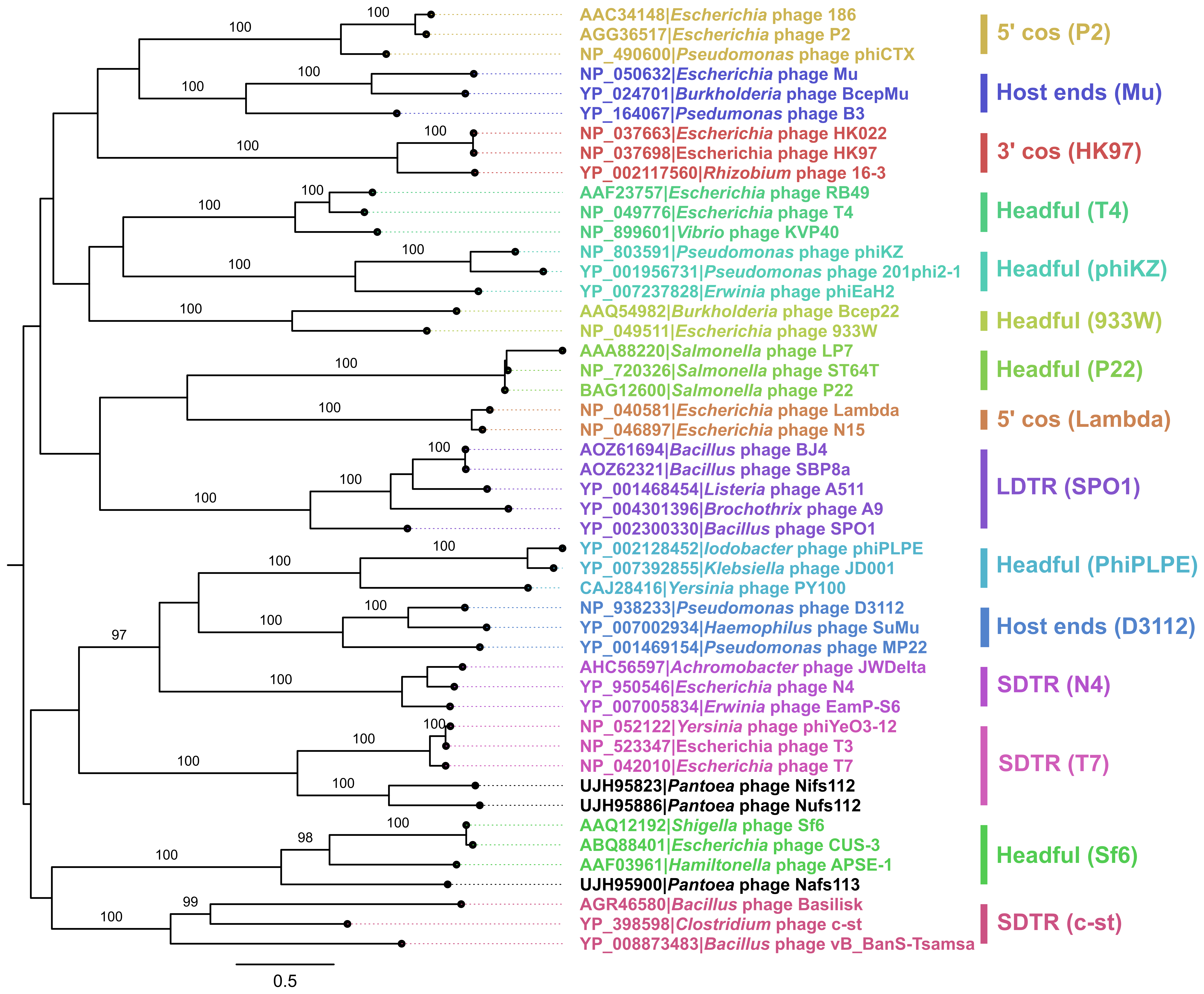

Supplement: Supplementary file 1 [file ijms-24-01820-s001.zip › SupplementaryFigureS1.png]

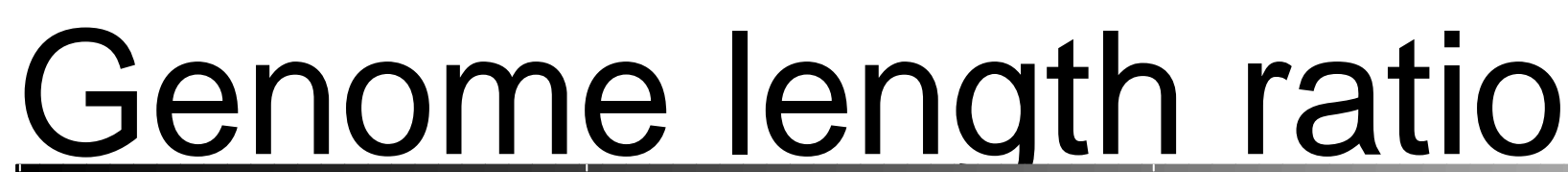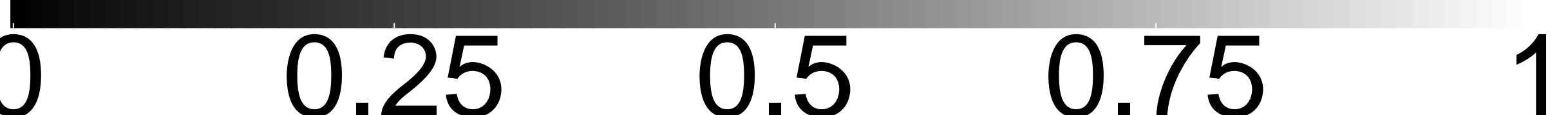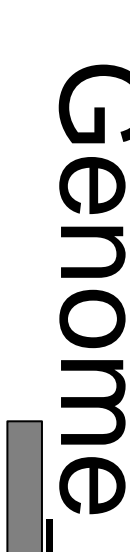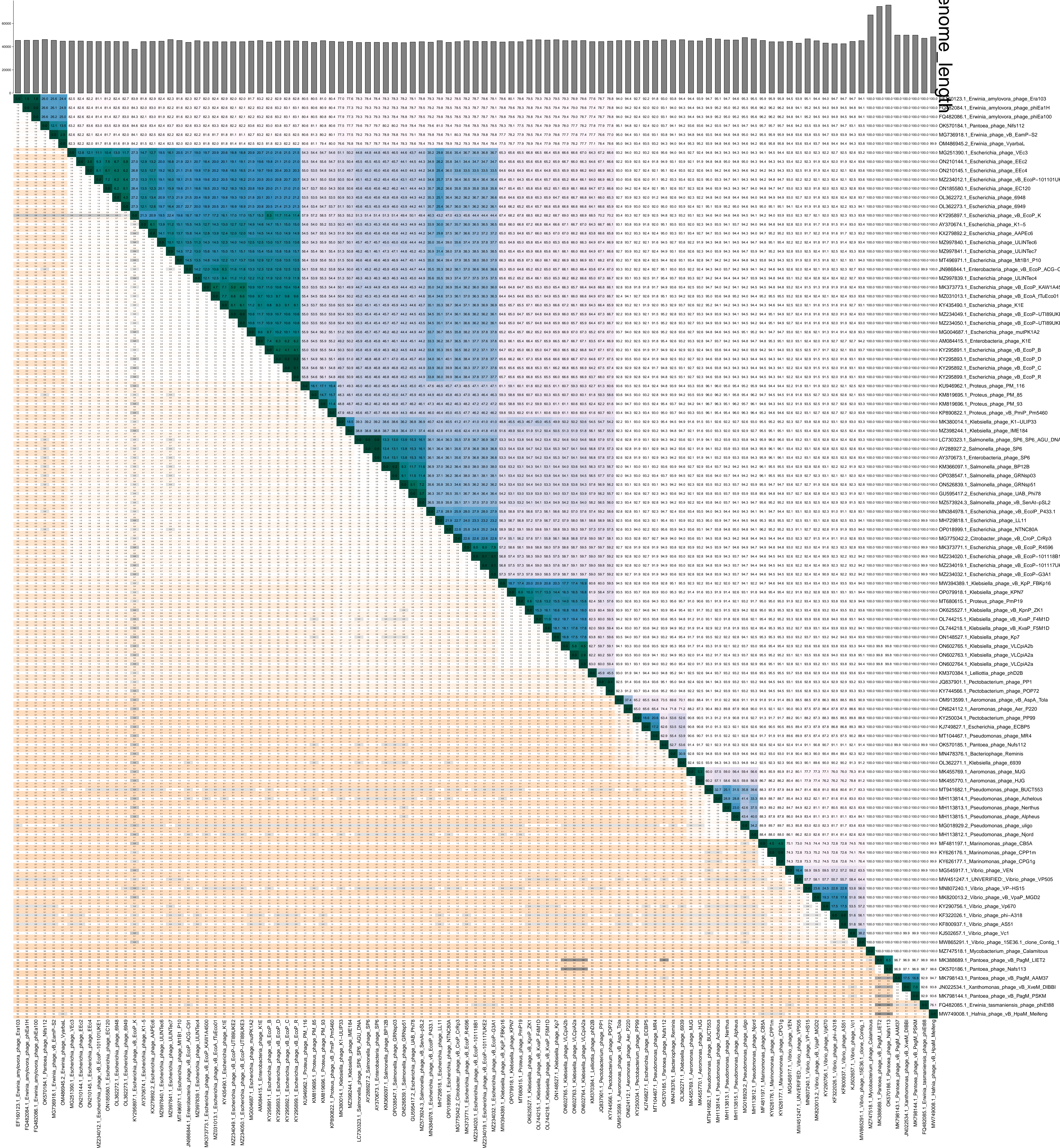

Supplement: Supplementary file 1 [file ijms-24-01820-s001.zip › SupplementaryFigureS2.PDF]

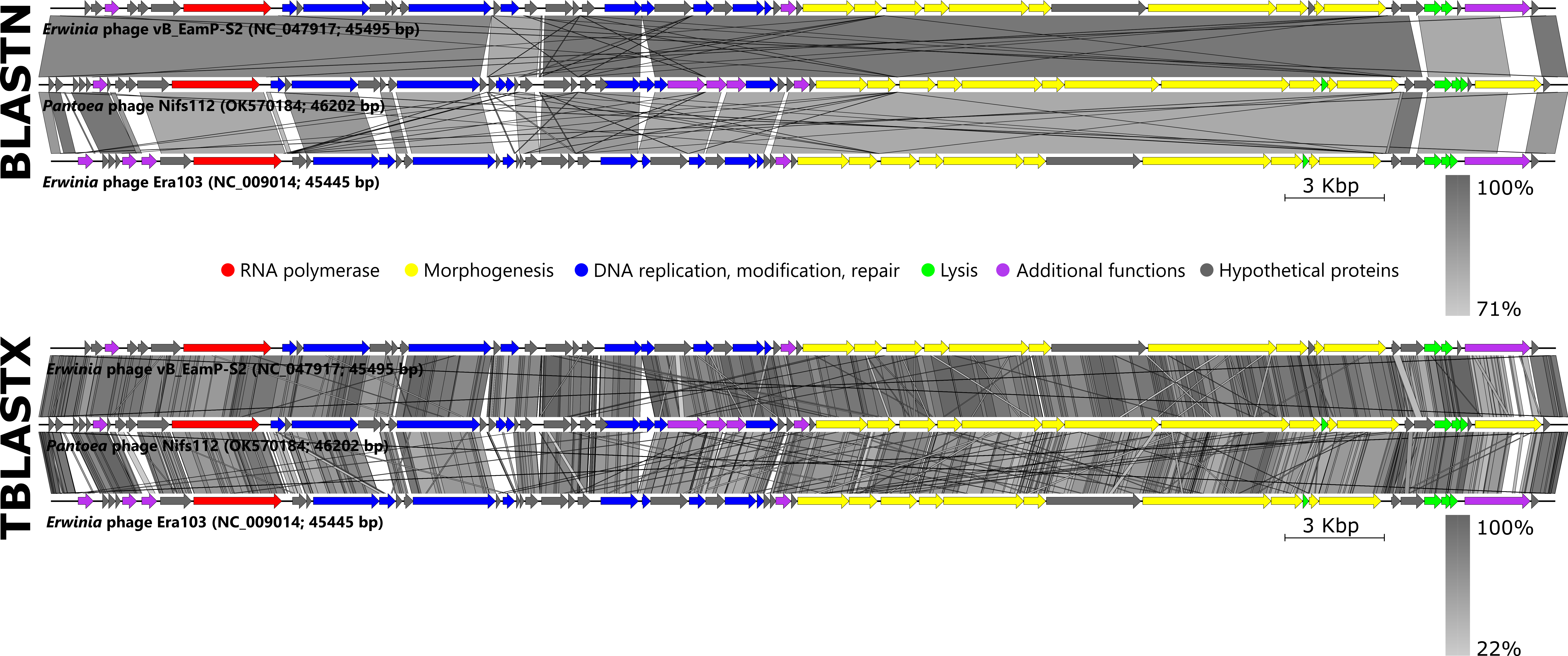

Supplement: Supplementary file 1 [file ijms-24-01820-s001.zip › SupplementaryFigureS3.png]

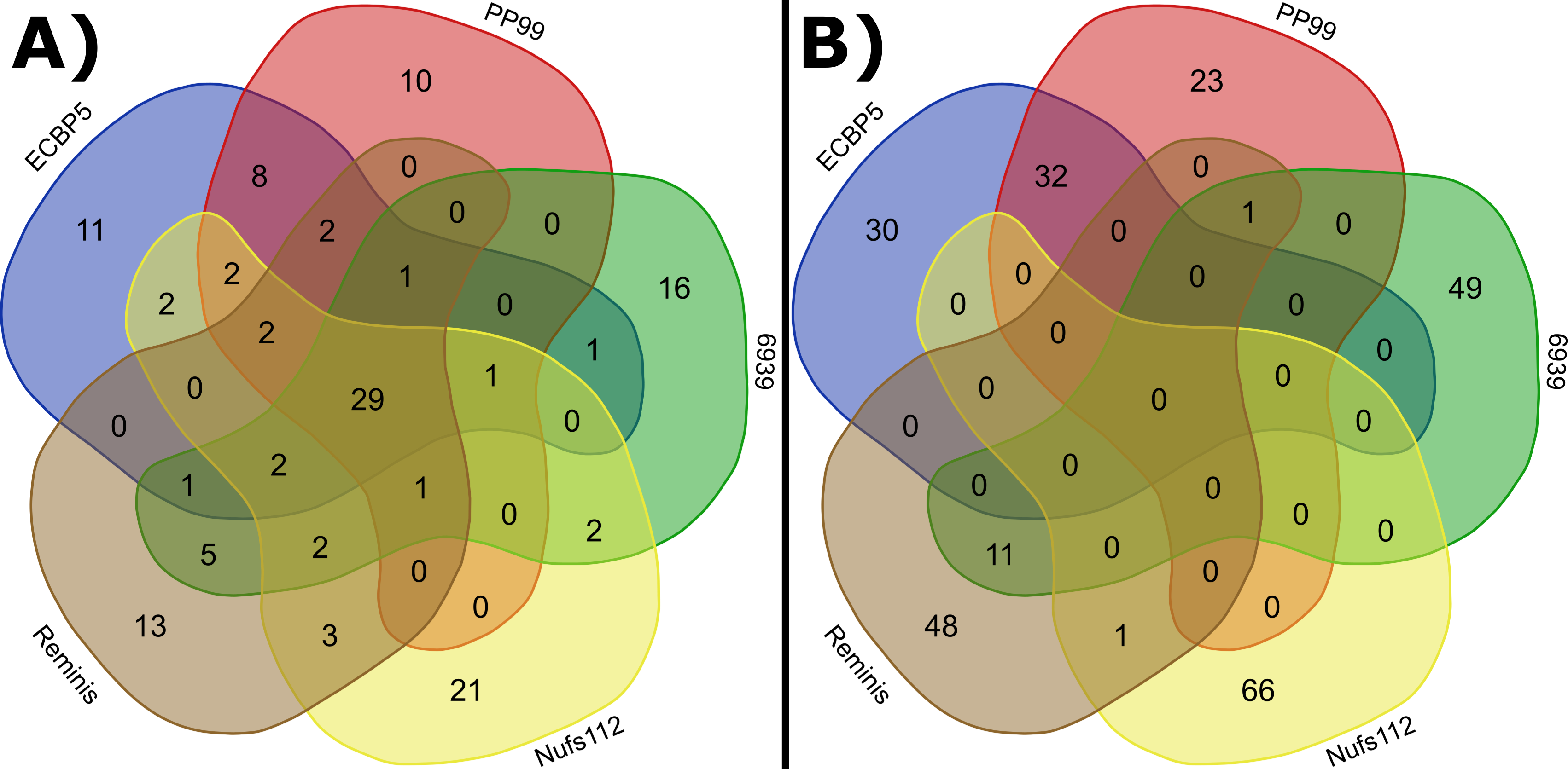

Supplement: Supplementary file 1 [file ijms-24-01820-s001.zip › SupplementaryFigureS4.png]

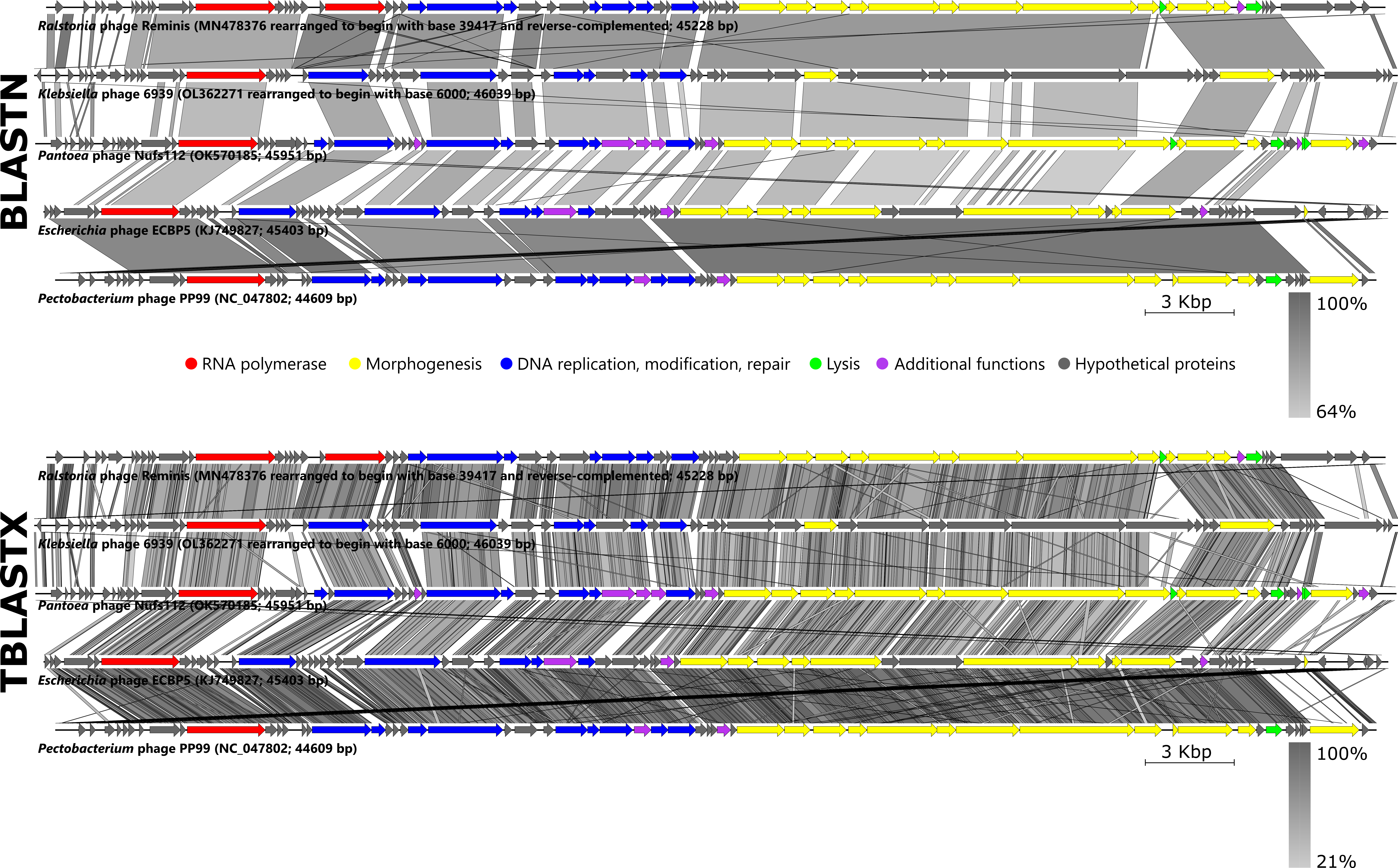

Supplement: Supplementary file 1 [file ijms-24-01820-s001.zip › SupplementaryFigureS5.png]

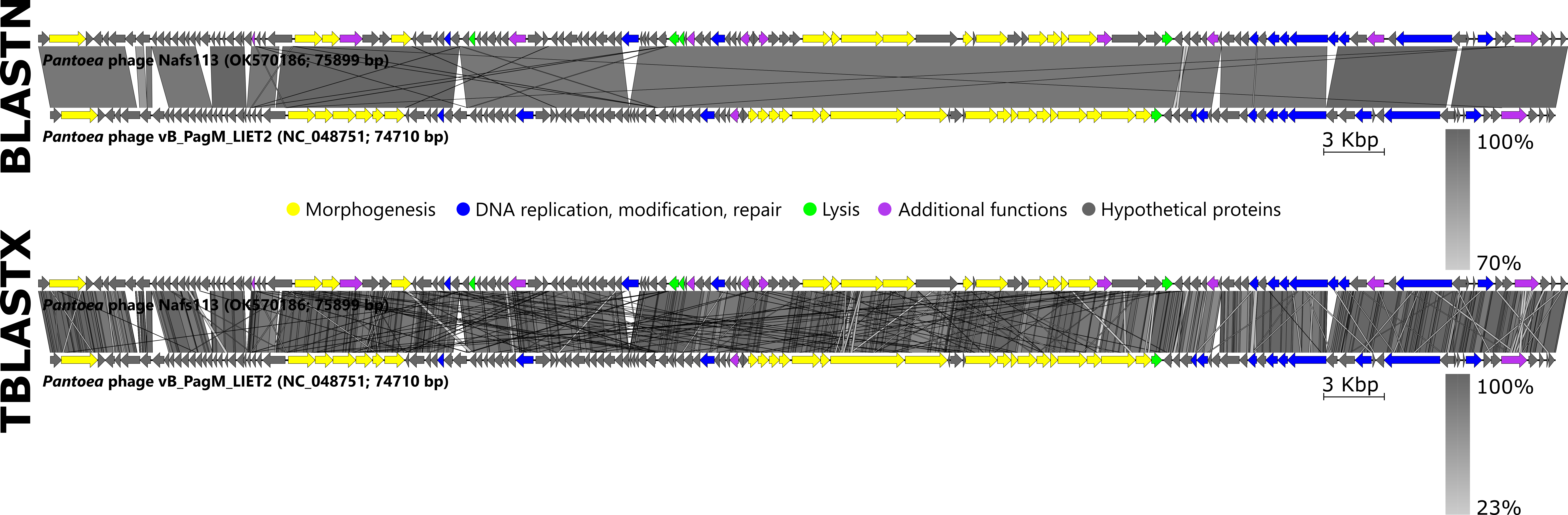

Supplement: Supplementary file 1 [file ijms-24-01820-s001.zip › SupplementaryFigureS6.png]
